# “I Look in Your Eyes, Honey”: Internal Face Features Induce Spatial Frequency Preference for Human Face Processing

**DOI:** 10.1371/journal.pcbi.1000329

**Published:** 2009-03-27

**Authors:** Matthias S. Keil

**Affiliations:** Basic Psychology Department, Faculty for Psychology, University of Barcelona, Barcelona, Spain; University College London, United Kingdom

## Abstract

Numerous psychophysical experiments found that humans preferably rely on a narrow band of spatial frequencies for recognition of face identity. A recently conducted theoretical study by the author suggests that this frequency preference reflects an adaptation of the brain's face processing machinery to this specific stimulus class (i.e., faces). The purpose of the present study is to examine this property in greater detail and to specifically elucidate the implication of internal face features (i.e., eyes, mouth, and nose). To this end, I parameterized Gabor filters to match the spatial receptive field of contrast sensitive neurons in the primary visual cortex (simple and complex cells). Filter responses to a large number of face images were computed, aligned for internal face features, and response-equalized (“whitened”). The results demonstrate that the frequency preference is caused by internal face features. Thus, the psychophysically observed human frequency bias for face processing seems to be specifically caused by the intrinsic spatial frequency content of internal face features.

## Author Summary

Imagine a photograph showing your friend's face. Although you might think that every single detail in his face matters for recognizing him, numerous experiments have shown that the brain prefers a rather coarse resolution instead. This means that a small rectangular photograph of about 30 to 40 pixels in width (showing only the face from left ear to right ear) is optimal. But why? To answer this question, I analyzed a large number of male and female face images. (The analysis was designed to mimic the way that the brain presumably processes them.) The analysis was carried out separately for each of the internal face features (left eye, right eye, mouth, and nose), which permits us to identify the responsible feature(s) for setting the resolution level, and it turns out that the eyes and the mouth are responsible for setting it. Thus, looking at eyes and mouth at the mentioned coarse resolution gives the most reliable signals for face recognition, and the brain has built-in knowledge about that. Although a preferred resolution level for face recognition has been observed for a long time in numerous experiments, this study offers, for the first time, a plausible explanation.

## Introduction

In the brain, the structure of neuronal circuits for processing sensory information matches the statistical properties of the sensory signals [1]. Taking advantage of these statistical regularities contributes to an “optimal” encoding of sensory signals in neuronal responses, in the sense that the code conveys the highest information with respect to specific constraints [2]–[6]. Among the various constraints which were formulated we find, for example, keeping metabolic energy consumption as low as possible [7]–[9], or keeping total wiring length between processing units at a minimum [10], or maximizing the suppression of spatio-temporal redundancy in the input signal [2], [11]–[14].

As for visual stimuli, natural images reveal (on the average) a conspicuous statistical regularity that comes as an approximately linear decrease of their (logarithmically scaled) amplitude spectra as a function of (log) spatial frequency [15]–[17]. This means that pairs of luminance values are strongly correlated [18], and this property could be exploited for gain controlling of visual neurons. Then, visual neurons would have equal sensitivities or response amplitudes independent of their spatial frequency preference [16]. According to this *response equalization hypothesis*, gain should thus be incremented with increasing spatial frequency, such that the distribution of response amplitudes of frequency-tuned neurons to a typical natural image is flat.

An argument in favor of employing response equalization (“whitening”) is that it would lead to an improvement of information transmission from one neuronal stage to another, because the output of one stage would match the limited dynamic range of a second one [19].

The present article builds upon previously reported results for whitened amplitude spectra of face images [20]: the whitened spectra reveal a spatial frequency maximum at 10–15 cycles per face, but only if external face features (such has hair) are suppressed. The predicted frequency maximum nevertheless agrees well with numerous psychophysical experiments, which found that face identity is preferably processed in a narrow band (bandwidth ≈2 octaves) of spatial frequencies from 8 to 16 cycles per face [21]–[29].

Despite of it all, the results presented in [20] indicate that the maxima in the amplitude spectra are caused by the compound effect of *horizontally* oriented internal face features (eyes, mouth & nose). Quantitatively, the maxima thus occur in units of “cycles per face *height*”, whereas most psychophysical studies instead measure their results in terms of “cycles per face *width*”. Furthermore, although a clear enhancement of horizontal amplitudes could be observed in the spectra, horizontal amplitudes showed a somewhat “noisy” dependence on spatial frequency. Both effects are a consequence of that face features were not considered individually, what causes a mixing of the spatial frequency content of individual face features in the spectra. The mixing leads to averaging-out effects such that any possible enhancement of spectral amplitudes at other than the horizontal orientation goes unnoticed, but also may cause interference effects which lead to the mentioned noisy dependence of amplitudes on spatial frequency.

The present study addresses the two issues by means of an extensive analysis of face images by means of Gabor filters. The filters were thereby parameterized (according to [30]) to match the spatial receptive field of band-limited, oriented and contrast sensitive neurons in the primary visual cortex [31]–[34]. (These cortical neurons are referred to as simple and complex cells, cf. [35]–[37]). Great care has been taken to guarantee the correct alignment of filter responses with respect to the position of internal face features (left eye, right eye, nose and mouth) prior to their averaging. Doing so permits to precisely elucidate how the frequency dependence of Gabor responses (and specifically the predicted frequency maxima) is related to each of the four internal face features.

The resulting graphs of whitened Gabor amplitudes versus spatial frequency are smooth and reveal distinct maxima at nearly all orientations. The most stable maxima, however, are observed at horizontal feature orientations in the first place, but also at vertical orientations. This observation holds true for all of the internal face features (even for the nose). The present study therefore shows how the individual internal face features contribute to the psychophysically observed frequency preference, and proposes concrete mechanisms of how higher amplitudes of whitened cell responses at an early level could possibly lead to the psychophysically measured effects.

## Methods

### Face Images

For the present study, 868 female face images and 868 male face images were used (*Face Recognition Grand Challenge* database FRGC, http://www.frvt.org/FRGC or www.bee-biometrics.org) [38]. Original images (1704×2272 pixels, 24-bit true color) were adjusted for horizontal alignment of eyes, before they were down-sampled to 256×256 pixels and converted into 8-bit grey-scale. Positions of left eye, right eye, and mouth [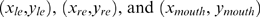, respectively] were manually marked by two persons (M.S.K. and E.C.) with an *ad hoc* programmed graphical interface. The face center position (≈nose) was approximated as 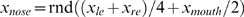 and 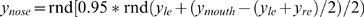, where 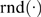 denotes rounding to the nearest integer value.

**Figure 1:**
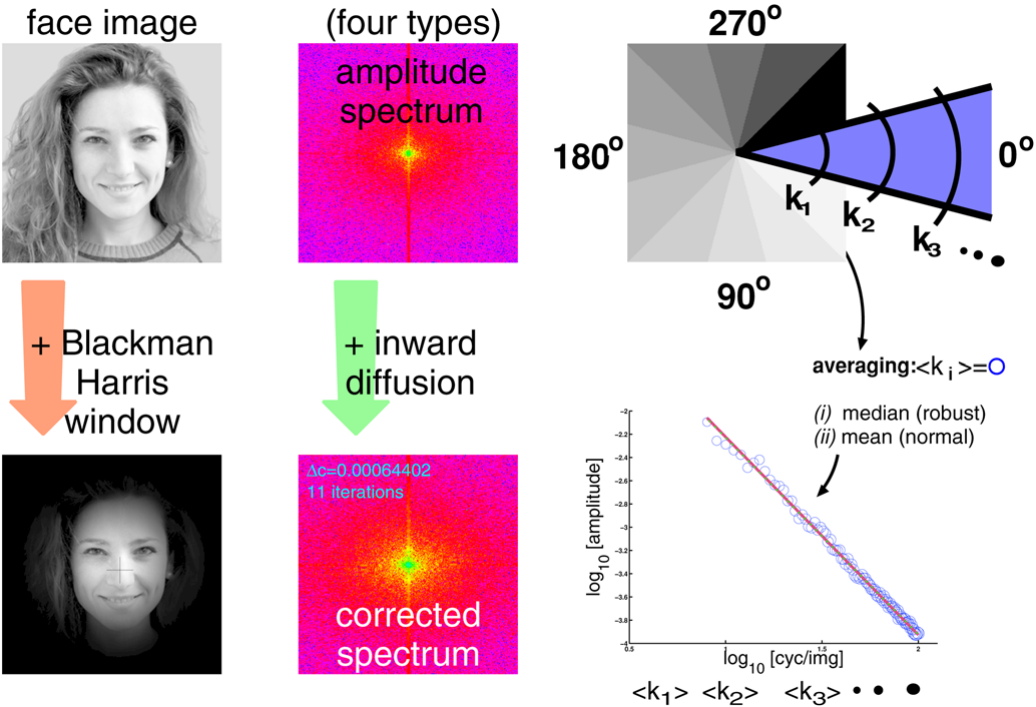
Computing slope values. The sketch summarizes the main steps that were taken in [20] for computing the slope values. Four types of spectra were considered (yielding four respective sets of slope values): *(i)* spectra of the original face images ( = raw), *(ii)* raw spectra corrected for truncation artifacts with *inward diffusion* ( = corrected raw), *(iii)* spectra of minimum 4-term Blackman-Harris (B.H.) windowed face images to suppress external face features [90], *(iv)* B.H. spectra corrected for the spectral “fingerprint” left by the application of the Blackman-Harris window. Now, in order to compute oriented slopes, a spectrum was subdivided into 12 pie slices (denoted by different shades of gray in the last image in the top row). Spectral amplitudes with equal spatial frequencies lie on arcs in the spectrum (schematically indicated by 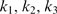). Amplitudes on arcs were averaged, either by “normal” statistical measures (i.e., location = mean & spread = standard deviation), or by outlier-insensitive “robust” measures (median & median absolute deviation MAD). Averaging yields a one dimensional (1-D) isotropic spectrum at each orientation (bottom right). A line with slope 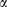 was then fitted to the double logarithmic representation of the 1-D spectra.

**Figure 2:**
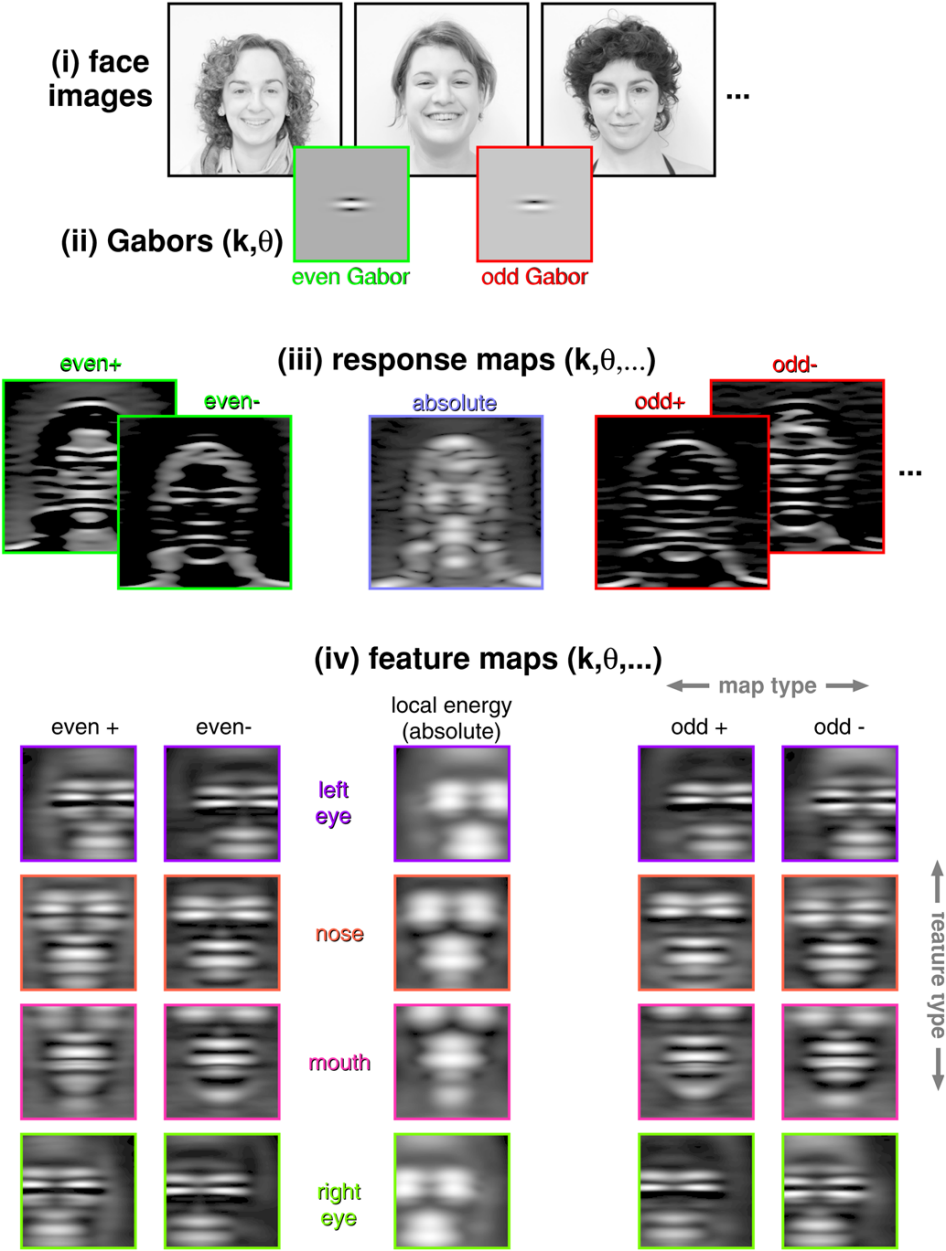
From images to feature maps. Illustration of the various steps involved in processing the face images *(i)* (size 256×256 pixels) with Gabor wavelets of orientation 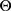 and spatial frequency 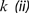 *(ii)*, where five response maps *(iii)* (size 256×256 pixels) are obtained at each 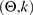. Response maps are subsequently centered at the four feature positions and cropped as illustrated with Figure 3. The aligned and cropped maps are averaged, giving rise to corresponding feature maps *(iv)* (size 127×127 pixels). Feature maps are parameterized by feature (4 possible values), gender (2), spatial frequency (39), orientation (12), and response type (5), what amounts to a total of 18720 feature maps.

**Figure 3:**
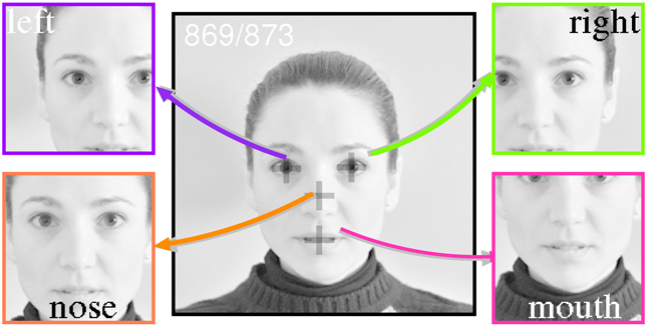
Alignment of face features. This figure illustrates the alignment procedure with a face image (note that this procedure is actually used for aligning response maps, cf. previous figure). Four subregions are extracted from each face image as shown, such that the corresponding feature of interest (left eye, right eye, nose, or mouth) is in the image center. Feature coordinates are indicated by crosshairs in the big ( = original) image, and were obtained through manual marking.

Due to copyright issues it was not possible to include original sample images from the FRGC database in this paper. The persons that are shown in Figures 1, 2, and 3 are surrogate images that were taken in the style of the database images. The depicted individuals gave their expressive permission to publish their photographs. Sample images from the FRGC database are shown in Figure 3 of [39], and in the supplementary material of [20].

### Dimension of Spatial Frequency

For conversion of spatial frequency units, face dimensions were manually marked with an *ad hoc* programmed graphical interface. The factors for multiplying “cycles per image” to obtain “cycles per face width” were 0.41±0.013 (females, 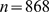) and 0.43±0.012 (males, 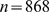). Corresponding factors for obtaining “cycles per face height” were 0.46±0.021 (females) and 0.47±0.018 (males). Conversion factors oblique orientations were calculated under the assumption that horizontal and vertical conversion factors define two main axis of an ellipse. Pooling of results over gender implied also a corresponding averaging of conversion factors.

### Slopes of Amplitude Spectra

The amplitude spectra of face images fall approximately linear as a function of frequency when both variables are scaled logarithmically [20]. Each amplitude spectrum was subdivided into 12 pie slices (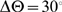) for computation of oriented spectral slopes 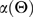 (Figure 1). A straight line with slope 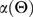 was fitted within the spatial frequency range from 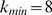 to 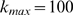 cycles per image to each pie with orientation 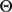. We used the function “robustfit” (linear regression with less sensitivity to outliers) provided with Matlab's statistical toolbox (*Matlab* version 7.1.0.183 R14 SP3, *Statistical Toolbox* version 5.1, see www.mathworks.com). In total, four amplitude spectra were considered (see Figure 1 & [20] for further details).

### Modeling Simple and Complex Cells

A 2-D-Gabor wavelet transform was used as a simplified model of V1 visual processing [16], [32], [34], [40]–[42]. Let 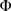 denote the spatial frequency bandwidth in octaves and 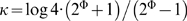. Let 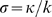, where 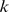 denotes spatial frequency in units of cycles per image. Let 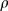 be the phase shift of each of the components of the pair of Gabor filters (the phase shift 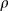 is not a relative phase shift: choosing 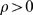 makes both the even and the odd Gabor wavelet shift by 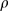, and does not affect their *relative* phase, i.e. they maintain their quadrature relationship). Let 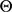 be an rotation angle in units of degrees. Then, in Fourier space, a constrained Gabor wavelet 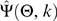 with spatial frequency 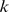 and orientation 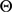 is defined as
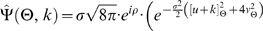
(1)

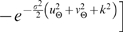
(2)(Convention: 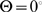 means that the wave vector points to the east, cf. Figure 1; 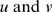 are frequency coordinates). Real and complex Gabor wavelets were parameterized to fit the receptive field data of even and odd simple cells, respectively [30] (spatial frequency bandwidth 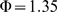 octaves [41],[43], orientation bandwidth 30 degrees [44],[45], aspect ratio 1.5 [41],[43],[46], and 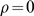 without loss of generality). Notice that here 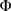 is constant (such that wavelets are self-similar with scale) whereas neuronal bandwidths generally *decrease* with the logarithm of 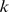. Gabor wavelets integrated to zero (admissibility constraint). Simple cell responses were taken as the rectified amplitudes of Gabor wavelets (positive even, negative even, pos. odd, neg. odd). Complex cell responses were computed with the *contrast energy* [16] or *local energy* [47],[48] model. Convolutions were performed in the Fourier space. We considered wavelet responses at spatial frequencies from 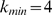 to 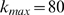 cycles per image, with increments 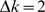 cycles per image. With this value of 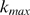, the maximum amplitude of the impulse response function was about two orders of magnitude higher than the spurious high frequency ripples that resulted as a consequence of filter truncation.

### Compacting Feature Maps

**Figure 4:**
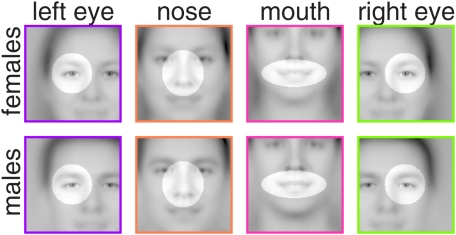
Regions of interest (ROIs). The regions over which representative feature map values were computed by spatial averaging (“ROI-compacting”) are highlighted in averaged face images. Note that the face images are shown only to illustrate ROI locations, since representative values were computed from feature maps. (Note furthermore that “full compacting” involves averaging across the entire feature map). For each feature type, a ROI thus defines a suitable set of spatial indices 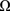, which contained 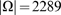 points (left and right eye), 2511 (mouth), and 2687 (nose), respectively, of a total of 127×127 feature map positions. The ROIs were selected manually. Identical ROIs were used for both gender.

In order to make the evaluation of results tractable, each (average) feature map was represented by a single scalar value (“compacted”), called *feature map amplitude*. This value is usually the spatial average. Spatial averaging could either take place over all feature map positions, or over feature-map-specific regions of interest as depicted in Figure 4 (“ROIs”). The overall predictions with respect to whitened feature map amplitudes remain similar if feature maps were compacted differently, for example by taking the maximum value, or by computing the average of only those values which exceed a given threshold value.

## Results

### Overview and Nomenclature

**Figure 5:**
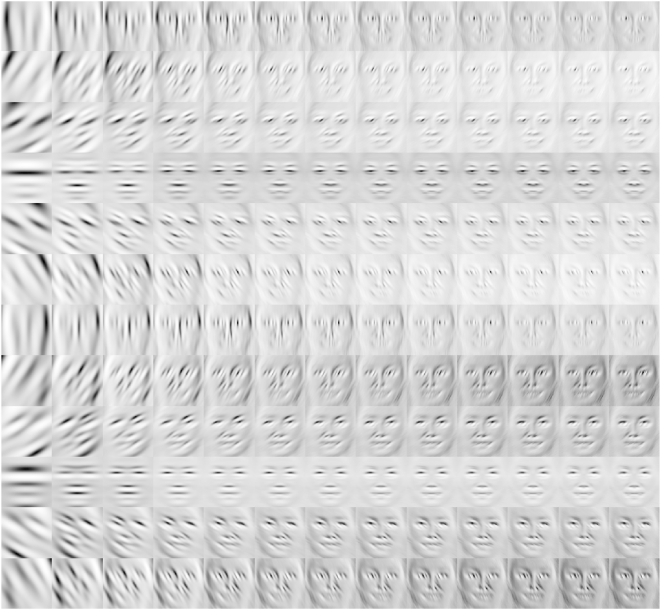
Odd feature maps. The display shows feature maps 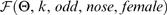, with response types *positve odd* (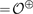) and *negative odd* (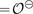) being displayed simultaneously according to 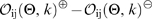. Brighter grey levels correspond to 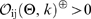, and darker grey levels correspond to 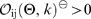. Thus, the mid grey level of each feature map indicates the zero response level. Each image represents the average of 868 response maps centered at the position of the nose. Along rows, orientation 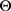 varies from top 0° to bottom 330° in steps of 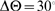. Along columns, the spatial frequency 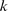 increases from left 8 to right 80 cycles per image in steps of 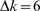 cycles per image, thus showing only 13 of a total of 39 spatial frequencies that were used in the analysis. For displaying, each feature map was normalized individually in order to improve the overall view.

Because the analysis is intricate at first sight, this section summarizes the main concepts and terms. The analysis takes the following steps. *First*, slopes of amplitude spectra are computed ( = spectral slopes 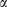). To this end four different types of amplitude spectra were considered, giving rise to four respective sets of slope values (summarized in Figure 1, see methods section). (A set of slope values contains the spectral slopes computed at different orientations). *Second*, each face image is projected on Gabor filters at different scales and orientations (Figure 2). Each projection results in a new “image” that is composed of a filter's response at the corresponding position of the face image. This filtered image defines a *response map* at a certain spatial frequency 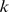 and orientation 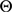. Five different types of response maps are distinguished: two with even symmetry, two with odd symmetry, and one combination involving both symmetries (more details are given below). *Third*, response maps are aligned according to the position of internal face features (left eye, right eye, mouth or nose – see Figure 3) and subsequently averaged. The averaged response maps are called *feature maps* (Figure 5). Each feature map 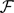 is parameterized by 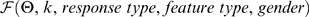. *Fourth*, the feature maps are response equalized (“whitened”) by using the spectral slopes at corresponding orientations. *Fifth*, to facilitate the analysis (18720 feature maps with 127×127 values each), each whitened feature map is *compacted* such that it is represented by a single scalar value ( = *feature map amplitude*). Compacting is carried out by computing the spatial average across the entire map (*full compacting*), or just over a small region around a feature of interest (*ROI-compacting*). The *regions of interest* (“ROIs”) are shown in Figure 4.

### Response Whitening

**Figure 6:**
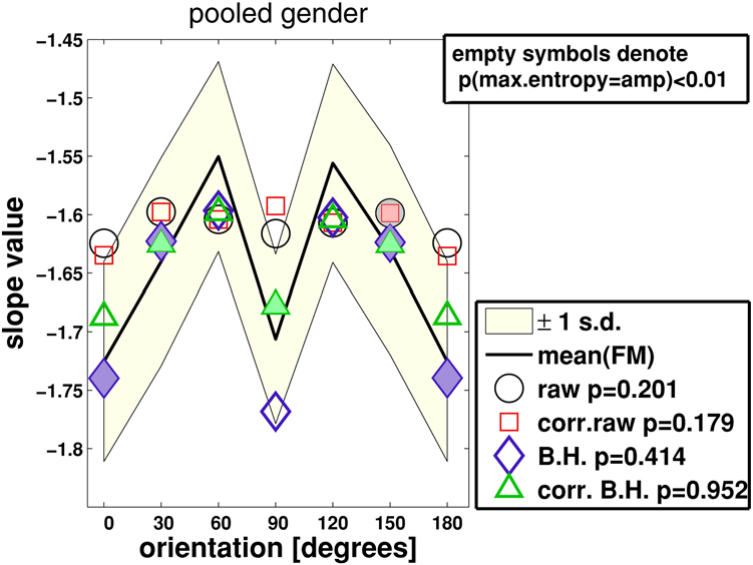
Slopes. Symbols denote oriented spectral slopes 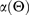 from the four amplitude spectra (circles = raw, squares = corrected raw, diamonds = Blackman-Harris (B.H.), and triangles = corrected B.H. – see methods section and Figure 1). The solid curve centered in the light-colored area denotes *maximum entropy slopes* 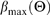 of feature map amplitudes (label “mean (FM)”). The light-colored area indicates ±1 standard deviation. Open symbols indicate where spectral slopes and maximum entropy slopes are significantly different from each other (one-way ANOVA at each orientation, 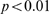). Filled symbols denote the opposite case (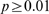). A further ANOVA test served to compare whether orientation-averaged slope values were drawn from the same underlying distribution. The respective probabilities are 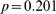 (raw spectrum versus maximum entropy slopes), 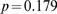 (corrected raw), 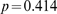 (B.H.), and 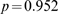 (corrected B.H.). Notice that slope values in the angular domain from 180° to 360° are equivalent to those being shown.

Oriented spectral slopes 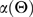 from the amplitude spectra were used to adjust the response gain ( = whitening) of Gabor filters [49]. The symbols in Figure 6 indicate the four sets of 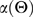. Gabor filters were parameterized such that they matched the spatial receptive fields of simple and complex cells in the primary visual cortex (see methods section). Cell responses (“response maps”) 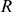 to a face image 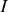 (size 256×256 pixels) were simulated by projecting the image onto a wavelet 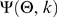 with spatial frequency 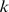 and orientation 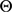, that is 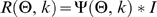 (convolutions were carried out in the Fourier domain, see equation 1 in the methods section). Response maps are complex-valued images with the same size as the face images. Cell types were distinguished by five corresponding response map types. Specifically, simple cell responses were taken as the rectified amplitudes of Gabor wavelets (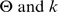 are omitted): positive even 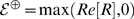 (with *Re*[^.^] denoting the real part) and negative even 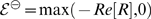. Positive and negative odd responses 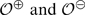, respectively, are defined analogously as the imaginary part 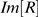. Complex cell responses 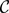 were computed with the *local energy* model [16],[48]: 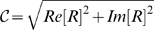.

To compute average cell responses over face images, each response map was centered in turn at the positions of the left eye, right eye, mouth and nose (internal face features, Figure 3), symmetrically cropped, and then summed separately for each of the four features. In this way, four types of so-called *feature maps* (size 127×127 pixels) were obtained for each of the five response maps, with 39 spatial frequencies 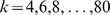 cycles per image, and at 12 orientations 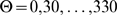 degrees (Figures 2 and 5).

Now, to test whether the response equalization hypothesis could account for face perception data, feature maps 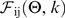 were whitened by multiplying each position 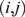 with 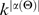, that is 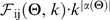 (with 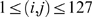) [50].

All in all we are left with four feature maps for each gender ( = 2), response type ( = 5), orientation ( = 12), and spatial frequency ( = 39). Each feature map in turn is composed of the responses of 127×127 model cells. This amounts to a data load of 18720×16129 (feature maps×values). To reduce this data load, each whitened feature map was represented by a single scalar value ( = *feature map amplitude*). This representative value was computed by either computing the average of response magnitudes over all 127×127 feature map positions (“full compacting”), or only over a region of interest that contained a single internal face feature (“ROI compacting”, Figure 4). A *response distribution* (or response curve) is then defined by considering feature map amplitudes as a function of 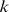 at some orientation 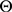.

If, as a result of whitening, response distributions were completely flat, we would not have gained any new insight. Therefore, we expect that the response distributions reveal residual structures as a function of 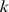 (ideally unimodal), which could be linked to face perception data.

### Response Distributions

**Figure 7:**
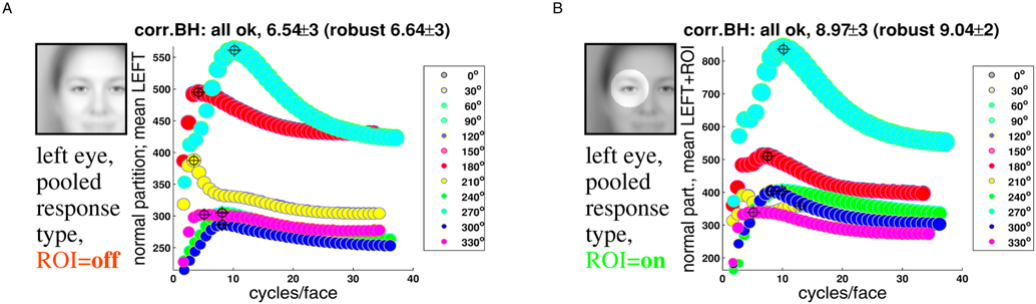
Response distributions with and without ROI. Both plots show pooled feature map amplitudes vs. spatial frequency (“response distribution”) for the left eye. Corrected Blackman-Harris slopes of individual face images were averaged by computing a *mean* slope value (as opposed to computing the median), which was used for whitening in this figure (see Figure S1 for whitening with uncorrected B.H. slopes). Line fitting was carried out by “*normal*” averaging of spectral amplitudes with equal frequencies (as explained in Figure 1). Response distributions were pooled across gender (male, female) and response type (positive even, negative even, positive odd, negative odd, local energy). Symbol sizes were scaled in proportion to standard deviations ( = overall standard deviation from averaging response maps, compacting and subsequent pooling). The standard deviations (s.d.) are usually very high with maxima exceeding often 100% (see text for an explanation). (A) Compacting the full feature map (inset, “ROI = off”). Relative s.d. lie between 105.4% (smallest symbol size) and 156.9% (biggest symbol). (B) Compacting over the circular region highlighted in the inset (“ROI = on”). Relative s.d. were 93.2% (minimum) and 142.1% (maximum). Crosshairs “⊕” indicate *valid maxima* (summarized in Figure 8). The average spatial frequency (±1 s.d.) of the valid maxima is shown at the top of each figure (robust: median ±1 MAD). Notice that mathematically curves at orientations 0°,30°,60°,90°,120°,150° are equivalent to the respective curves in the angular domain from 180° to 330°. However, numerical errors (especially due to sampling artifacts associated with the convolution kernels) can cause small deviations. The relative absolute deviations (mean ±1 s.d. in %) are 0.26±0.15 (normal partition; maximum 0.52%) and 0.15±0.15 (robust partition; maximum 0.46%).

Figure 7A (and corresponding Figures S1, S2, S3, S4) shows response distributions at different orientations for full compacting. Response distributions (“curves”) for different response types and gender were pooled together for compiling these figures.

The curves are not flat, but all have maxima (*valid maxima* are indicated by encircled black crosshairs). The *average* spatial frequency (±s.d.) of the valid maxima in Figure 7A is 6.54±3 cycles per face (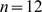 orientations). Observe that the maxima of response distributions at horizontal feature orientations (90° and 270°, turquoise curves) are always situated at ≈10 cycles per face, irrespective of feature type. Specifically, the “horizontal” curves vary by far less than the others as a function of ROI. Furthermore, curves at horizontal and nearby oblique orientations (±30°) also reveal the most pronounced deviation from a flat response distribution. Notice that horizontally oriented Gabor filters match the orientations of eyes, mouth and the nose bottom.

Upon introducing a ROI, the response curves at horizontal feature orientations are shifted upwards relative to the curves at remaining orientations. This effect is particularly striking when comparing response distributions at horizontal and vertical orientations, where “horizontal” curves are getting enhanced relative to “vertical” curves with “ROI = on”. Often, curves that coincide with feature orientations revealed also clearer maxima in the sense that the maxima were lifted with respect to smaller values (Figure 7B & corresponding panels in Figures S1, S2, S3, S4). In contrast, curves at oblique orientations (e.g., 150°) sometimes get flatter and/or reveal multi-modal distributions.

Especially interesting in this context is to consider the response distributions for the nose (Figure S4): Here, the up-shifting of the “horizontal” curve relative to the “vertical” one is the smallest (compared to the rest of features), and the “vertical” curve is showing a more pronounced maximum then. A consistent interpretation of this behavior is that the nose has of course an important vertical orientation component (the bridge of the nose), whereas with eyes and mouth vertical orientations are less important. Nevertheless, as with the other features, also the nose has its most “important” orientation component situated *horizontally* (the bottom termination). Furthermore, the spatial frequency maximum of the bridge of the nose is smaller than the maximum of the “horizontal” curve.

### Standard Deviations

The standard deviations of the pooled data were computed from three components: *(i)* averaging the aligned response maps to compute feature maps, *(ii)* compacting the feature maps to obtain feature map amplitudes, and finally *(iii)* pooling feature map amplitudes. High standard deviations are produced *(i)* because of the variation between individual face images, and *(ii)* because Gabor wavelets produce responses to face images with only a few wavelets generating relatively high responses (sparse responses: [16]).

Standard deviations always decreased upon using a ROI for two reasons. First, secondary features that appear beside of the feature of interest in the center are cropped (cf. insets in Figure 7), and the variation around the aligned features is smallest between face images. Second, high Gabor wavelet responses occur mainly to the feature of interest. As a consequence, peak feature map amplitudes with ROI are bigger than without, because the relative amount of small-valued Gabor responses is smaller within a ROI.

### Valid Maxima

**Figure 8:**
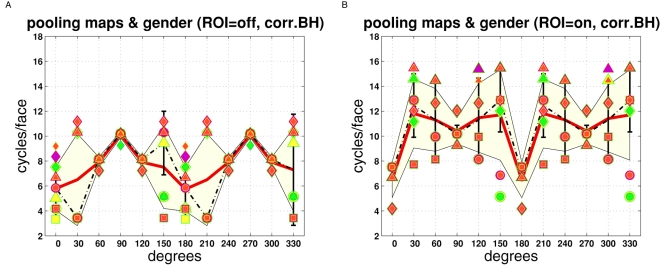
Summarising the maxima of response distributions. Data points indicate spatial frequencies associated with a peak of a response distribution curve (“valid maximum”). Whitening was performed with slopes from the corrected Blackman-Harris amplitude spectra. Oriented spectral slopes were either computed according to the *(i)* normal partition (amplitude-averaging according to mean) or the *(ii)* robust partition (median). The spectral slopes in turn were either averaged by *(iii)* computing their mean value 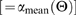 or by *(iv)* computing their median 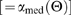. Symbol colors (and sizes) indicate corresponding combinations: *yellow* = normal partition & mean of slopes (i.e.,*i* together with *iii*), *violet* = robust & mean, *green* = normal & median, and *red* = robust & median. Symbol shapes, on the other hand, denote the different features: ○ = left eye, □ = right eye, ◊ = mouth, and 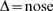. The mean value (median value) of the data points at each orientation is indicated by the solid red line (dash dotted line), with the shaded area indicating ±1 standard deviation. The error bars denote a robust estimate of standard deviation (by means of median absolute deviation MAD) with respect to the median value ( = dash dotted line). (A) “ROI = off”, pooling together response type and gender. (B) Same as with *a*, but for “ROI = on”.

Here, the behavior of the spatial frequency maxima of the response distributions ( = valid maxima) is summarized. Upon introducing a ROI, the great majority of the maxima shifted to higher spatial frequencies (e.g., Figure 7A: from 6.54±3 cycles per face without ROI to 8.97±3 with ROI). As already mentioned, most of the maxima which did not shift at all were those at horizontal orientations. Valid maxima of response distributions are summarized in Figure 8 and Figures S5, S6, S7, S8, respectively, with juxtapose data for “ROI = off” and “ROI = on”. The up-shifting-effect of spatial frequency maxima can be clearly seen in these figures, with valid maxima associated with ROI-compacting being situated at around 10 cycles per face.

The results discussed so far were obtained with the mean spectral slopes 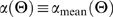. In order to probe the robustness of the predicted spatial frequency maxima, a further set of slope values were considered for whitening, that is the median 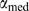 of individual slope values (remember: one slope value per face image). Whitening with 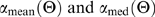 led to similar predictions for the spatial frequencies of the maxima at virtually all orientations (see corresponding colors in Figure 8 and Figures S5, S6, S7).

### Bandwidths

**Figure 9:**
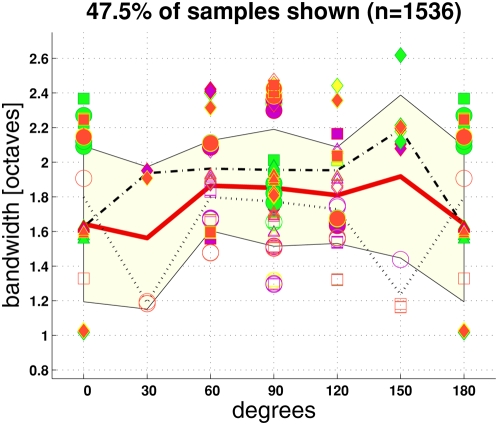
Estimating bandwidths of response distributions. Estimated full bandwidths at half height 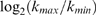 (in octaves) of response distributions, where gender and response type were pooled. Each response distribution curve was considered individually. A bandwidth estimate (“sample”) was proposed by the computer, which had to be accepted or rejected by user interaction. Less than half of the curves had a shape which allowed for a reasonable estimation (47.5% of 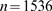 curves), with most samples at 90°. Bandwidths from 210° to 360° are equivalently to the corresponding shown bandwidths (0 to 180°). This fact was exploited to remove inconsistently accepted or rejected bandwidth estimates, as user interaction proceeded across the full angular domain. The red curve shows the mean of all samples at an orientation. The lightly colored area indicates ±1 standard deviation. The dash-dotted line is the mean of the samples with “ROI = on” (filled symbols), the dotted line for the “ROI = off” samples (open symbols). Symbol shape denotes feature type as with the previous figure, and symbol color denotes spectra: *yellow* = raw, *violet* = corr.raw, *green* = B.H. & *red* = corr.B.H. The partition/slope-averaging combinations listed in the previous figure (items *i* to *iv*) are not further distinguished here, meaning that the same symbols were used for all of these combinations.

For a subset of all response distributions it was possible to estimate spatial frequency bandwidths (Figure 9): “ROI = off” had a greater variation of bandwidths than “ROI = on”. With “ROI = off”, most of the bandwidth estimates lie between 1 and 2 octaves. With “ROI = on”, bandwidth showed a tendency to increase on the average, with the majority of the bandwidth estimates lying in the range from 1.6 to 2.4 octaves. These estimated bandwidths are in good agreement with the psychophysically predicted bandwidths for face processing.

### How White Is White? Maximum Entropy Slopes

As the function for whitening 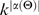 was parameterized with slopes computed from the the different types of amplitude spectra, I asked whether these slopes indeed produced the “most whitened” response distributions. Accordingly, another set of slope values 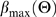 was computed as follows. Feature maps were compacted without previous whitening. Whitening rather was iteratively performed through gain adjustment of feature map amplitudes – by multiplication with 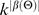. For each 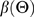 from −2.5 to −1, the degree of whitening was quantified in steps of 0.01 by computing the Shannon entropy [51] of the whitened response distributions. Maximally white response distributions are associated with a maximum in entropy at 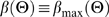 (*maximum entropy slope*).

Figure 6 juxtaposes averaged maximum entropy slopes (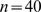 samples for each 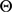) with averaged amplitude spectrum slopes (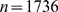). Averaging took place over all parameters but orientation.

Maximum entropy slopes achieve the best agreement with the corr.B.H.-slopes, both when averaging over orientations (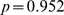), and when evaluating statistical significance at each orientation separately (filled symbols indicate 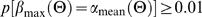).

In comparison with the B.H. data, slopes from the raw spectra have the worst agreement with the maximum entropy slopes. This discrepancy is ascribable to external face features: Slopes were computed individually for each face image, and external face features like the hairline could influence individual slopes directly in the raw and corr.raw spectra. By contrast, in the feature maps the external features are averaged out and partially cropped (fully cropped with ROI). The mismatch between using slopes (raw & corr.raw) with external face features being present in order to whiten feature maps that are nearly devoid of the external features causes corresponding response distributions to be not “optimally” white.

## Discussion

Here, I studied whitened and averaged responses of Gabor filters to large number of face images (whitening refers to response equalization). Gabor filters were parameterized as to match spatial receptive field properties of simple and complex cells in the cortex (see methods), and averaging was *feature-specific* (Figure 3). The results obtained here extend the predictions of a previously conducted analysis (ref. [20]) of averaged and whitened amplitude spectra in three important ways. *(i)* The use of Gabor wavelets permitted the examination of the orientation dependence of spatial frequency predictions, whereas in the previous study only an amplitude enhancement at horizontal orientations was revealed. *(ii)* Averaging of Gabor response maps was done according to features (yielding corresponding feature maps), whereas the spatial frequency content of internal face features was mixed in the previous study (“mixing” occurs because Fourier spectra do not retain absolute spatial information explicitly). Mixing caused interference effects and averaging-out of any amplitude enhancement at others than the horizontal orientation. *(iii)* The previous study showed a somewhat noisy dependence of the spatial frequency versus amplitude curve, due to mixing effects. The response amplitude curves shown here are in contrast very smooth.

For the whitening procedure, the slopes of four different types of amplitude spectra were considered (Figure 1), in order to probe robustness of predictions. The slopes obtained from the corrected-Blackman-Harris-window spectrum (corr. B.H.) were thereby the closest to a flat response distribution in the sense that they best maximized Shannon entropy (cf. maximum entropy slopes, Figure 6).

As a consequence of whitening, most response distributions ( = compacted feature maps) were not flat or “white” (Figure 7), but revealed unimodal distributions irrespective of their orientation, with maxima centered at around 8–12 cycles per face when compacted with a feature-specific region of interest (ROI), and somewhat lower without it (≈4–10 cycles per face, Figure 8).

Responses at horizontal feature orientations were scarcely affected by employing a ROI: their maxima did not shift significantly, and curve shape did not alter either (Figure 7, turquoise curve). This behavior stands in contrast to response distributions at oblique feature orientations, which showed the strongest changes. Estimated bandwidths of the response distributions were about 1.6 to 2 octaves with ROI. Somewhat smaller bandwidth estimates were obtained without ROI (Figure 9).

Feature maps (Figure 2) were obtained by properly centering Gabor response maps at feature positions prior to averaging the latter (Figure 3). In this way external face features (e.g., hair) and uncentered features were averaged out (since they varied strongly between face images), while centered features were kept well focused (Figure 4). The unfocused features correspond to low spatial frequencies, what generates maxima at lower spatial frequencies than with ROI.

The ROI versus no-ROI data therefore demonstrate that higher responses are obtained by filters matching the orientation and spatial frequency of internal face features. The results furthermore suggest an orientation dependence of preferred spatial frequencies, similar to the oblique effect (e.g., [52]–[54], but see [55]): Horizontal and vertical oriented features have more ecological “importance” than features at oblique orientations.

Several psychophysical studies suggest that recognition of face identity works *best* in a narrow band (bandwidth about 2 octaves) of spatial frequencies from ≈8 to ≈16 cycles per face [21]–[25],[27],[56],[57]. Notice that this does not mean that face recognition exclusively depends on this frequency band, as faces can still be recognized when corresponding frequency information is suppressed [27],[29]. In addition, it seems that observers can specifically attend to the spatial frequencies that support recognition (“diagnostic spatial frequencies”), and that the allocation of attended frequencies can be altered in a task-specific fashion [58],[59]. Hence, observers could intentionally attend to other than the preferred spatial frequencies if the latter frequencies are not available, but the non-preferred frequencies may be associated with a reduced signal-to-noise ratio (e.g., in terms of class separation [39]) and/or may imply a corresponding increase in time for completing a successful face recognition [29].

The preferred spatial frequencies for face recognition are not significantly affected by the structure of the background on which a face does appear [60], so the results presented here are unlikely to be specific for the considered set of face images.

How can higher response amplitudes be linked to an enhanced perceptual sensitivity for face identification? The proposed whitening mechanism implies that neural populations which encode a natural scene at an instant in time adapt in order to match the statistics of the input such as to similar sensitivities are established for neurons with different spatial frequency selectivity (response equalization). A flat or white distribution of responses is also compatible with the notion of sparse coding. For face images, we saw that a completely flat distribution could not be obtained (at least with the proposed mechanism), and that the flattest possible distributions rather were unimodal (in most cases). As we could readily interpret the distribution as being proportional to the underlying probability distribution, the brain could increase processing speed for face recognition if it “looked” first at those spatial frequencies which occur more often. If these frequencies are removed (as it happened in some of the mentioned psychophysical experiments), then the brain has to actively examine other spatial frequencies to complete a successful recognition, what would yield to an increase in recognition time. A corresponding increase in recognition time has indeed been observed experimentally [29].

Also from an biophysical point of view, the whitened response distributions could translate into a decreased processing time. In the response-equalized population of neurons, higher response amplitudes (which occur at around 10 cycles per face) are associated with shorter response latencies. Or, more specifically, if we assume that whitening changes synaptic efficiency, then neurons tuned to 10 cycles per face will reach spiking threshold faster because they are driven by higher post-synaptic currents, and thus corresponding information could in principle arrive earlier at successive face recognition stages.

The *critical retinal illumination* is the transition luminance between deVries-Rose [61],[62] and Weber's law, describing the increasing and the saturating part, respectively, of the human contrast sensitivity function. (The transition luminance is described by the van Nes-Bouman law [63]). Interestingly, this critical retinal illumination was found to vary with 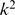 for foveally viewed cosine gratings [64]. This result permits to derive an explicit expression for the neural modulation transfer function (MTF) of the visual pathway [65], with a linear dependence of the MTF on 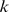. So, could whitening of face images be conveyed by the neural modulation transfer function? Amplitude spectra of natural images vary approximately with 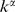, where 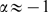 [15]–[17], but for our face images 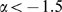 (Figure 6; [20]). Thus, the MTF could in principle carry out a pre-whitening of spatial frequency channels, leaving some residual whitening to the specific neural systems for face processing (according to 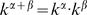). Notice, however, that whitening with 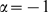 produces a smaller number of valid spatial frequency maxima in the response distribution curves (without ROI: 8%, with ROI: 71%), and these maxima underestimate the psychophysically found frequencies (without ROI≈3, with ROI≈4 cycles per face).

What about other stimulus classes? A comparison can be readily drawn between the perception of letters and faces. Letter identification has been found to be sensitive to spatial frequencies of about 3 cycles per letter height, e.g., [66]–[69]. Similar to the present study and ref. [20], Põder performed an analysis of letter power spectra (i.e., the squared amplitude spectra; [70]). He subdivided power spectra into annuli that were one octave wide, and then integrated power across each annulus. This procedure yielded an energy maximum at 2–3 cycles per letter, consistent with psychophysical results and an interpretation of the maximum in terms of letter stroke frequency [71].

Faces and letters are examples of relatively “constrained” objects: Characters printed on a paper are two-dimensional objects which do not reveal additional information when the paper is rotated in three dimensional space. Similarly, we usually see upright faces in our visual field, and face recognition performance decreases significantly with inverted faces [72],[73]. It seems that this drop in recognition performance is associated with corresponding changes in face processing strategies. In brief, upright faces seem to undergo an increasingly holistic or configural processing in the brain (i.e., in terms of relationships between internal face features or *face parts*, respectively), as opposed to inverted faces, e.g., [74]–[79]. It has been proposed that inverted faces are processed in a similar way as arbitrary objects (but see, e.g., [80] or [81] for a discussion). Indeed, there is evidence for part-processing at early stages for face processing (e.g., [80],[82] with references), and it appears that the familiarity with a face modulates the degree to which configural processing is evoked over part-based processing ([83],[84] including references).

The findings of the present study relate best to early face processing, and specifically to part-based processing (ROI versus no-ROI). In this context, it is interesting that the N170 or M170 response (an early face-selective response which is observed in electro- or magnetoencephalography data, respectively) can be evoked by the presence of isolated internal face features, especially the eyes [85],[86]. This result is consistent with the present data, where all internal face features induced distinct spatial frequency maxima.

Further evidence supports the notion that the eye region is especially important for face identification [87], and that subjects use the same spatial frequencies for identifying upright and inverted faces [57]. The latter result can be interpreted such that the frequency preference for face recognition indeed reflects properties of early and part-based face processing.

Different spatial frequency bands were nonetheless found to support part-based and configural face processing, respectively ([88] - but see [81]). For instance, matching performance with configural changes was found to be superior for low-pass filtered faces [89] (cut off ≈8 cycles per face width), whereas for detecting differences between internal face features, high-pass filtered faces (>32 cycles per face width) seem to give a better performance. The results here bear some loose similarity with this notion in two ways.

*First*, the ROI versus no-ROI data revealed that feature-specific results with “ROI = on” yielded slightly higher spatial frequency predictions than the whole-face condition “ROI = off”. However, as discussed above, this frequency shift is a consequence of averaging feature-map amplitudes within a region around a feature of interest (“ROI = on”), versus averaging of feature map amplitudes unspecifically (“ROI = off”). The unspecific averaging includes both the feature of interest (well focused), and secondary features and external face features, which appear unfocused or blurry (Figure 4), thus introducing low spatial frequency content which, upon averaging feature map amplitudes (“compacting”), causes the observed frequency shift.

*Second*, predicted spatial frequencies were higher at horizontal (90°, 270°) than at vertical orientations (0°, 180°), and predicted spatial frequencies increased relatively more upon applying a ROI at vertical orientations. (In contrast, horizontal Gabor filters match the orientations of internal face features, and consequently a ROI has only a smaller effect; oblique orientations reveal compound effects). The response distribution curves for vertical orientations (Figure 7) show similar magnitudes for “ROI = on” and “ROI = off”. Therefore, vertically oriented Gabor filters do not only pick up spatial frequency content of internal face features, but also an important part from the rest of the face. This suggests that vertical spatial frequency content may be better suitable for processing configural parts of the face, for example for measuring inter-ocular distance. Because the predicted frequencies at vertical orientations are lower than at horizontal orientations (both for “ROI = on” and “ROI = off”), this orientational effect resembles the aforementioned psychophysical findings which reported that part-based processing is supported by higher spatial frequencies than holistic processing.

How general are the results of the present study? Here it has been shown that the preferred spatial frequency band for human face recognition originates from internal face features, and that each of the internal features in isolation induces the same frequency preference. My result of course is rather invariant to inversion: the predicted spatial frequencies would not change if the study would have been conducted with a database of inverted faces. As aforesaid, a corresponding invariance has also been found by a recent psychophysical experiment: humans use the same spatial frequencies for recognition of upright and inverted faces [57]. What about horizontal head turning? Assume a moderate head turning such that internal face features remain visible. Then, a differential effect would occur for horizontally (90°) and vertically (0°) oriented spatial frequencies. Horizontal spatial frequency predictions can be expected to remain approximately constant, although response distribution curves may appear noisier. Vertical and oblique spatial frequency predictions, however, can be expected to reveal a stronger variation (this variation is suggested by comparing the ROI versus non-ROI data of the fronto-parallel case). Also, the magnitude and type of variation (for all orientations) may depend on the specific feature (eye, mouth, or nose), and the degree of head turning.

Recently, we were able to show that an enhanced class discrimination for face images is obtained at similar spatial frequencies which humans preferably use for face recognition [39]. This suggests that also artificial face recognition systems could exploit the spatial frequency dependency of face recognition in order to increase efficiency, either in terms of speed, accuracy, or memory economy. And it also suggests that humans may use this special range of spatial frequencies because it is best suited for distinguishing between different individuals.

## Supporting Information

Figure S1 More response distributions I (left eye, B.H. slopes). Same as Figure 7, but here for Blackman-Harris (i.e., not corrected) spectral slopes. **(a)** Compacting the full feature map with relative s.d. lying between 105% (smallest symbol size) and 156.8% (biggest symbol). **(b)** ROI-compacting with relative s.d. between 93.2% and 142.1%.
(0.11 MB EPS)

Figure S2 More response distributions II (right eye, corr. B.H.) Same as Figure 7, but here for the right eye. **(a)** no ROI, relative s.d. between 103.4% and 158.9%. **(b)** ROI-compacting, relative s.d. between 92.9% and 137.4%.
(0.10 MB EPS)

Figure S3 More response distributions III (mouth, corr. B.H.) Same as Figure 7, but here for the mouth. **(a)** no ROI, relative s.d. between 84% and 142.5%. **(b)** ROI-compacting, relative s.d. between 72.1% and 125.1%.
(0.10 MB EPS)

Figure S4 More response distributions IV (nose, corr. B.H.) Same as Figure 7, but here for the nose. **(a)** no ROI, relative s.d. between 106.2% and 148.3%. **(b)** ROI-compacting, relative s.d. between 82.8% and 116.7%.
(0.10 MB EPS)

Figure S5 Maxima of response distributions II (raw). Like Figure 8, but here for the raw slopes. **(a)** “ROI = off”, **(b)** “ROI = on”.
(0.06 MB EPS)

Figure S6 Maxima of response distributions III (corr. raw). Like Figure 8, but here for the corrected raw slopes. **(a)** “ROI = off”, **(b)** “ROI = on”.
(0.06 MB EPS)

Figure S7 Maxima of response distributions IV (B.H.). Like Figure 8, but here for the B.H. slopes. **(a)** “ROI = off”, **(b)** “ROI = on”.
(0.06 MB EPS)
